# Prognostic role of endometrial pattern compared with thickness and estradiol in euploid HRT-FET cycles

**DOI:** 10.3389/fmed.2026.1809726

**Published:** 2026-08-13

**Authors:** Nuri Peker, Ayşen Yücetürk, Esra Tığlı, Özge Karaosmanoğlu, Burak Elmas, İlke Özer Aslan, Bülent Tıraş

**Affiliations:** 1 Department of Obstetrics and Gynecology, Assisted Reproductive Technologies Unit, Acıbadem Maslak Hospital, İstanbul, Türkiye; 2 Department of Obstetrics and Gynecology, Kütahya Health Sciences University, Kütahya, Türkiye; 3 Department of Obstetrics and Gynecology, Acıbadem Mehmet Ali Aydınlar University, İstanbul, Türkiye

**Keywords:** endometrial pattern, endometrial thickness, estradiol, euploid blastocyst, frozen embryo transfer, progesterone

## Abstract

**Background:**

Do endometrial pattern types show an association with clinical outcomes in an IVF-ET cohort with pattern diversity, and do endometrial thickness and serum E_2_ and P_4_ levels measured on the day of endometrial assessment provide additional predictive value regarding reproductive outcomes?

**Methods:**

This retrospective HRT-FET study included 237 euploid blastocyst transfer cycles. The effects of endometrial pattern types (Type A–B–C) on pregnancy, miscarriage, and live birth were evaluated; the relationship between endometrial thickness and hormonal parameters and reproductive outcomes was analyzed; and predictive performance was assessed.

**Results:**

Significant differences were observed between endometrial pattern types for total pregnancy, biochemical pregnancy, miscarriage, and live birth rates (*p* = 0.004), with Type A showing the highest live birth rate. Endometrial thickness did not differ across pattern groups (*p* = 0.3819) or pregnancy outcome (*p* = 0.5676). Thickness categories showed significant distribution difference (*p* = 0.014), with higher live birth rates in >10.1 mm. ROC analyses indicated no predictive value for endometrial thickness in pregnancy (AUC = 0.505; cut-off 8.75 mm) or live birth (AUC = 0.5103; cut-off 11.85mm). E_2_ and P_4_ levels did not differ between pattern types (E_2_ *p* = 0.3819 and *p* = 0.2646; P_4_ *p* = 0.0799 and *p* = 0.2928), and ROC analyses for E_2_ showed limited predictive power in Type A (AUC = 0.5505; cut-off 279.5pg/ml) and Type B (AUC = 0.5382; cut-off 369.5pg/ml).

**Conclusions:**

Endometrial pattern may be associated with clinical outcomes, whereas endometrial thickness and serum E_2_ levels demonstrated limited discriminatory power. These findings highlight the heterogeneity in the existing literature and indicate that their role in decision-making during HRT-FET cycles should be reconsidered.

## Introduction

The prevailing view in reproductive medicine is that successful implantation depends on the precise synchronization between a developmentally competent embryo and a receptive endometrium (1, 2). Many studies have evaluated endometrial thickness and structure as the prognostic parameters for successful outcomes in *in vitro* fertilization-embryo transfer (IVF-ET). However, there is still no clear consensus on whether endometrial ultrasonographic characteristics can predict pregnancy outcomes (3).

One of the structural changes that occur in the endometrium is its thickening during the late proliferative phase of the menstrual cycle. During this phase, transvaginal ultrasound can identify the endometrium as having 3 distinct patterns: (1) A distinct central hypoechoic line bounded by two outer hyperechoic layers, defining a three-layered or “triple line” pattern; (2) an intermediate pattern where a three-layered appearance is present but is less distinct or mildly heterogeneous; and (3) a homogeneous hyperechoic pattern where the typical triple line structure is absent and the endometrium appears uniformly echogenic or irregular (4). It is known that these defined endometrial patterns have a significant effect on the relationship between endometrial thickness (EMT) and clinical pregnancy rate; yet, despite some studies suggesting that a trilaminar pattern may be associated with improved implantation and pregnancy outcomes, the evidence is inconsistent and heterogeneous across clinical settings (3, 5). For instance, it is demonstrated that although endometrial thickness > 8 mm and a triple-line pattern were associated with higher clinical pregnancy rates, these parameters failed to consistently predict live birth, highlighting the uncertainty surrounding their true prognostic value and need for further investigation (3, 5).

Thus, in this study we aimed to reassess the association between endometrial pattern types and reproductive outcomes, including biochemical pregnancy, miscarriage, and live birth in an IVF-ET cohort with pattern diversity. Secondary objectives included assessing whether endometrial thickness, examined as both a continuous variable and a percentile-based categorical measure, correlates with reproductive success, and determining whether serum estradiol (E_2_) and progesterone (P_4_) levels measured on the day of endometrial assessment provide additional predictive value. We hypothesized that endometrial morphology might exhibit some association with pregnancy outcomes, whereas hormonal parameters would demonstrate limited discriminatory power.

## Materials and methods

### Study design and patients

This retrospective study was conducted between January 1, 2020, and January 1, 2024 at the IVF center of Acibadem Maslak Hospital, a tertiary-level assisted reproduction unit. Study was conducted in accordance with the Declaration of Helsinki, and the study protocol was approved by the local review board and ethics committee of Acibadem University (Approval No: 2026-13/605). Written informed consent was obtained from the participants.

#### Inclusion and exclusion criteria

Women aged 20–45 years undergoing hormone-replacement frozen embryo transfer (HRT-FET) with a single euploid blastocyst confirmed as PGT-A normal were included in the study. Only cycles with an endometrial thickness greater than 7 mm on the day of progesterone initiation were eligible. Exclusion criteria included the presence of severe intrauterine adhesions, congenital uterine malformations (such as didelphys uterus or bicornuate/unicornuate uterus), or any structural pathology that could impair endometrial development. In addition, cycles with serum E_2_ levels below 150 pg/ml on the progesterone-starting day were excluded. Among 348 screened HRT-FET cycles with available Pre-implantation genetic testing for aneuploidy (PGT-A) results, 237 euploid blastocyst transfer cycles met the eligibility criteria and were included in the study.

#### IVF-ICSI/ vitrification and warming/embryo transfer

PGT- A was performed to all blastocysts before vitrification. Embryos graded (Gardner classification) as CC were excluded from the study and PGT-A was not performed to these embryos. Vitrification and warming were performed with Kitazato media (Japan) per manufacturer instructions. PGT-A normal, euploid embyos (s) were transferred under abdominal ultrasound guidance using a soft catheter. The inner tip was positioned in the upper uterine cavity, within 1 cm of the fundal apex, and the embryo (s) deposited gently.

#### Endometrial preparation

All patients underwent hormone-replacement frozen embryo transfer (HRT-FET) cycles. A GnRH agonist (Zoladex^®^, 3.6 mg; AstraZeneca, UK) was administered subcutaneously on day 21 of the preceding cycle for pituitary down-regulation. On cycle day 2 or 3, patients underwent a baseline transvaginal ultrasound. Endometrial preparation was initiated in those with an endometrial thickness ≤ 5 mm and no ovarian activity. Oral estradiol valerate (Estrofem^®^ 2 mg; Novo Nordisk, Denmark) was prescribed twice daily for 4 days, three times daily for the subsequent 5 days, and four times daily for an additional 4 days, for a total of 13 days of estradiol supplementation. This regimen resulted in a total estradiol dose of 8 mg/day prior to endometrial evaluation. Patients were then scheduled for a follow-up visit, during which endometrial thickness and pattern were assessed.

#### Endometrial assessment and grouping

**Figure 1:**
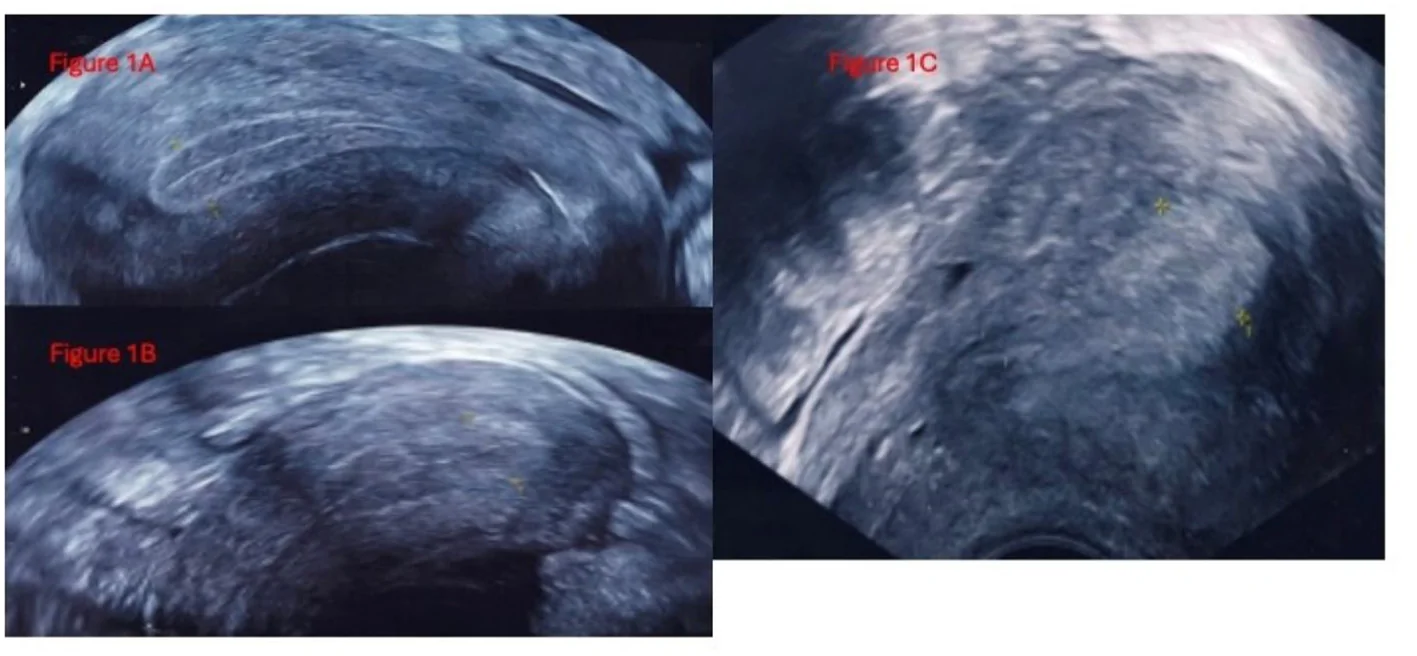
Transvaginal ultrasound images of endometrial pattern types. **(A)** Type A: A clear three-layered “triple-line” endometrium consisting of a central hyperechoic line within a homogeneous endometrium bounded by two hyperechoic peripheral layers. **(B)** Type B: A partially trilaminar but less distinct endometrium with an irregular or poorly defined central and/or outer line. **(C)** Type C: A uniformly hyperechoic endometrium without a visible triple-line structure.

Cycles with an endometrial thickness < 7 mm on the day of evaluation were deemed ineligible for embryo transfer and were canceled. Patients with an endometrial thickness ≥7 mm were further classified into three groups according to ultrasound pattern (Figure 1):

**Group (i) (Type A):** A clear three-layered “triple-line” endometrium consisting of a central hyperechoic line within homogeneous endometrium bounded by two hyperechoic peripheral layers (Figure 1A).

**Group (ii) (Type B):** A partially trilaminar but less distinct or mildly heterogeneous endometrium with an irregular or poorly defined central and/or outer line (Figure 1B).

**Group (iii) (Type C):** A uniformly hyperechoic endometrium without a visible triple-line structure and/or irregular, non-trilaminar pattern (Figure 1C).

Serum E_2_ and P4 levels were also measured on this day. Cycles exhibiting premature progesterone elevation (P4 >1.0 ng/ml) or insufficient estradiol levels (E_2_ < 150 pg/ml) were canceled.

Endometrial thickness categories were defined using the 33^rd^ and 66th percentile values of the sample distribution (P33 = 8.7 mm, P66 = 10.1 mm) rather than arbitrary clinical cutoffs, as no universally accepted threshold has been established in the literature for HRT-FET cycles.

#### Progesterone support and embryo transfer

Patients with appropriate hormonal levels were started on progesterone replacement therapy consisting of Prolutex 25 mg/subcutaneously twice daily and Progestan 200 mg vaginal capsules, two capsules three times daily. Embryo transfer was scheduled 6 days after initiation of progesterone therapy. On the day of embryo transfer, cycles with serum progesterone levels < 10 ng/ml were canceled. For patients with progesterone levels between 10–20 ng/ml, a single intramuscular (IM) dose of progesterone ((Progestan^®^ 50 mg; Koçak Pharma, Turkey) was administered in addition to routine luteal support. Cycles with progesterone levels ≥20 ng/ml proceeded without additional intervention and were included in the study.

#### Pregnancy assessment

Twelve days after embryo transfer, serum β-hCG levels were measured to confirm pregnancy.

The primary outcomes of the study were the effects of endometrial pattern on pregnancy, miscarriage and live birth rates. The secondary outcome of the study was relationship between endometrial thickness and E_2_, P_4_ levels on progesterone start day and pregnancy, miscarriage and live birth rate.

### Statistical analysis

Statistical analyses were performed using GraphPad Prism 8.0 (Graphpad Software, San Diego, CA, USA) and Jamovi (version 2.x). The distribution of continuous variables was assessed using the Shapiro–Wilk test, and non-normally distributed data were reported as median (min–max). Differences between endometrial pattern types and continuous variables were analyzed using the Kruskal–Wallis test; *post-hoc* pairwise comparisons were performed using the Fisher's exact test when necessary. Relationships between categorical variables were assessed using the chi-square test, and cell frequencies were interpreted for significant models. Endometrial thickness categories were created using the P33 and P66 percentile values according to the sample distribution. Additionally, a binary logistic regression analysis was performed to evaluate the independent effect of endometrial pattern type on live birth after adjusting for maternal age. Odds ratios (OR) with 95% confidence intervals (CI) were calculated. The omnibus likelihood ratio test was used to assess the overall significance of the predictors.

ROC curve analyses were performed to evaluate the predictive value of endometrial thickness and serum E_2_ levels in predicting pregnancy achievement and live birth; the area under the curve (AUC), 95% confidence intervals, sensitivity, and specificity were reported. *p* < 0.05 was considered statistically significant.

## Results

In this study, the effects of endometrial pattern types (Type A–B–C) on pregnancy outcomes in the study population were evaluated; the relationship between endometrial thickness and pregnancy, miscarriage, and live birth rates was analyzed in detail. Additionally, the distribution of demographic and hormonal parameters according to endometrial pattern groups was examined, and predictive performance was tested using ROC analyses.

The baseline demographic and clinical characteristics of the study population stratified by endometrial pattern type are summarized (Table 1).

**Table 1:** Baseline demographic characteristics of the study population according to the endometrial pattern types as median (min-max).

Parameter	Type A (*n* = 177)	Type B (*n* = 37)	Type C (*n* = 23)	*p* value
Age (years)	32.0 (21–40)	35.0 (23–40)	35.0 (25–40)	**0.0049**
BMI (kg/m^2^)	24.8 (17.6–39.0)	24.5 (18.1–34.0)	25.2 (18.0–34.9)	0.8749
Infertility duration (years)	5.0 (1–11)	6.0 (1–14)	7.0 (1–22)	0.2882

Values are presented as median (min–max). Comparisons between groups were performed using the Kruskal–Wallis test. Bold p-value indicates statistical significance (p < 0.05).

Baseline demographic characteristics of the study population according to endometrial pattern types are presented in Table 1. There was a statistically significant difference in age among the three groups (*p* = 0.0049), with the Type A group having a lower median age (32.0 years) compared to Type B (35.0 years) and Type C (35.0 years). Body mass index and duration of infertility did not differ significantly between the groups (*p* = 0.8749 and *p* = 0.2882, respectively), indicating that the groups were comparable in terms of these baseline characteristics. Given the significant age difference, this variable was considered as a potential confounder in the interpretation of reproductive outcomes.

To assess whether the observed age difference confounded the association between endometrial pattern and live birth, a binary logistic regression analysis was performed adjusting for maternal age (Table 2).

**Table 2:** Binary logistic regression analysis evaluating the independent effect of endometrial pattern type on live birth after adjusting for maternal age.

Variable	OR	95% CI	*p*
Age (per year)	0.94	0.89–1.00	0.062
Pattern Type (ref: Type A)	
Type B vs A	1.32	0.64–2.74	0.455
Type C vs A	10.82	3.04–38.51	< 0.001

Omnibus likelihood ratio test for pattern type: χ^2^ = 20.18, df = 2, *p* < 0.001. Model fit: AIC = 314, McFadden R^2^ = 0.066. OR > 1 indicates higher odds of not achieving live birth compared to the reference group.

A binary logistic regression analysis was performed to evaluate the independent effect of endometrial pattern type on live birth after adjusting for maternal age (Table 2). The overall model was statistically significant for endometrial pattern type (χ^2^ = 20.18, df = 2, *p* < 0.001). Type C endometrial pattern was associated with significantly higher odds of not achieving live birth compared to Type A (OR = 10.82, 95% CI: 3.04–38.51, *p* < 0.001), whereas no significant difference was observed between Type B and Type A (OR = 1.32, 95% CI: 0.64–2.74, *p* = 0.455). Maternal age showed a trend toward significance but did not reach the statistical threshold (OR = 0.94, 95% CI: 0.89–1.00, *p* = 0.062), suggesting that although increasing age may reduce the likelihood of live birth, this effect was not statistically significant after adjusting for endometrial pattern. These findings confirm that the association between endometrial pattern type and live birth is independent of maternal age.

**Figure 2:**
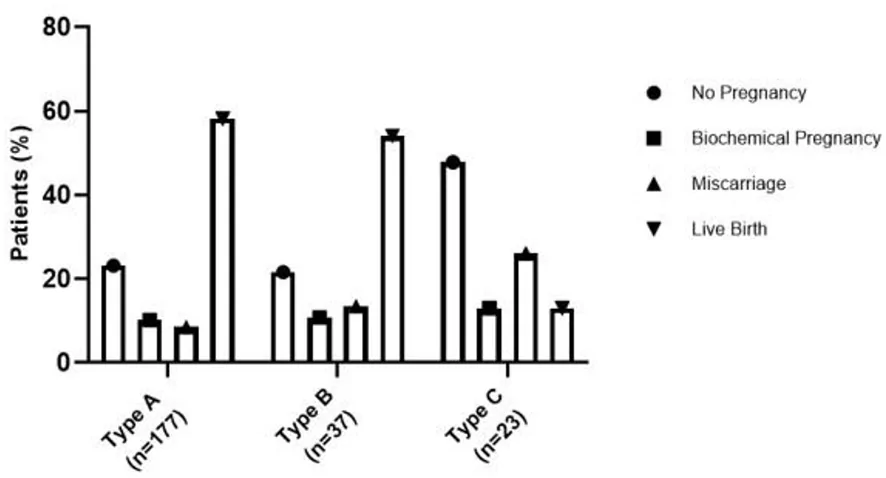
Distribution of pregnancy outcomes according to endometrial pattern types (Type A, Type B, and Type C). Pregnancy, biochemical pregnancy, clinical miscarriage, and live birth rates are presented for each endometrial pattern group. Bars display the comparative proportions across outcome categories.

The effect of endometrial pattern types (Type A, Type B, and Type C) on pregnancy outcomes was evaluated (Figure 2).

When comparing the distribution of pregnancy outcomes according to endometrial pattern types, a statistically significant difference was generally found between the groups (χ^2^ = 19.11, df = 6, *p* = 0.004). In pairwise comparisons with Fisher's exact test, no significant difference was observed in live birth rates between Type A and Type B endometrial patterns (58.19% and 54.05%, respectively; *p* = 0.716). In contrast, when comparing Type A and Type C endometrial patterns, a significantly higher live birth rate was found in the Type A group (58.19% and 13.04%, respectively; *p* < 0.0001). Similarly, when comparing the Type B and Type C groups, the live birth rate was found to be significantly higher in the Type B group (54.05% and 13.04%, respectively; *p* = 0.0023).

**Figure 3:**
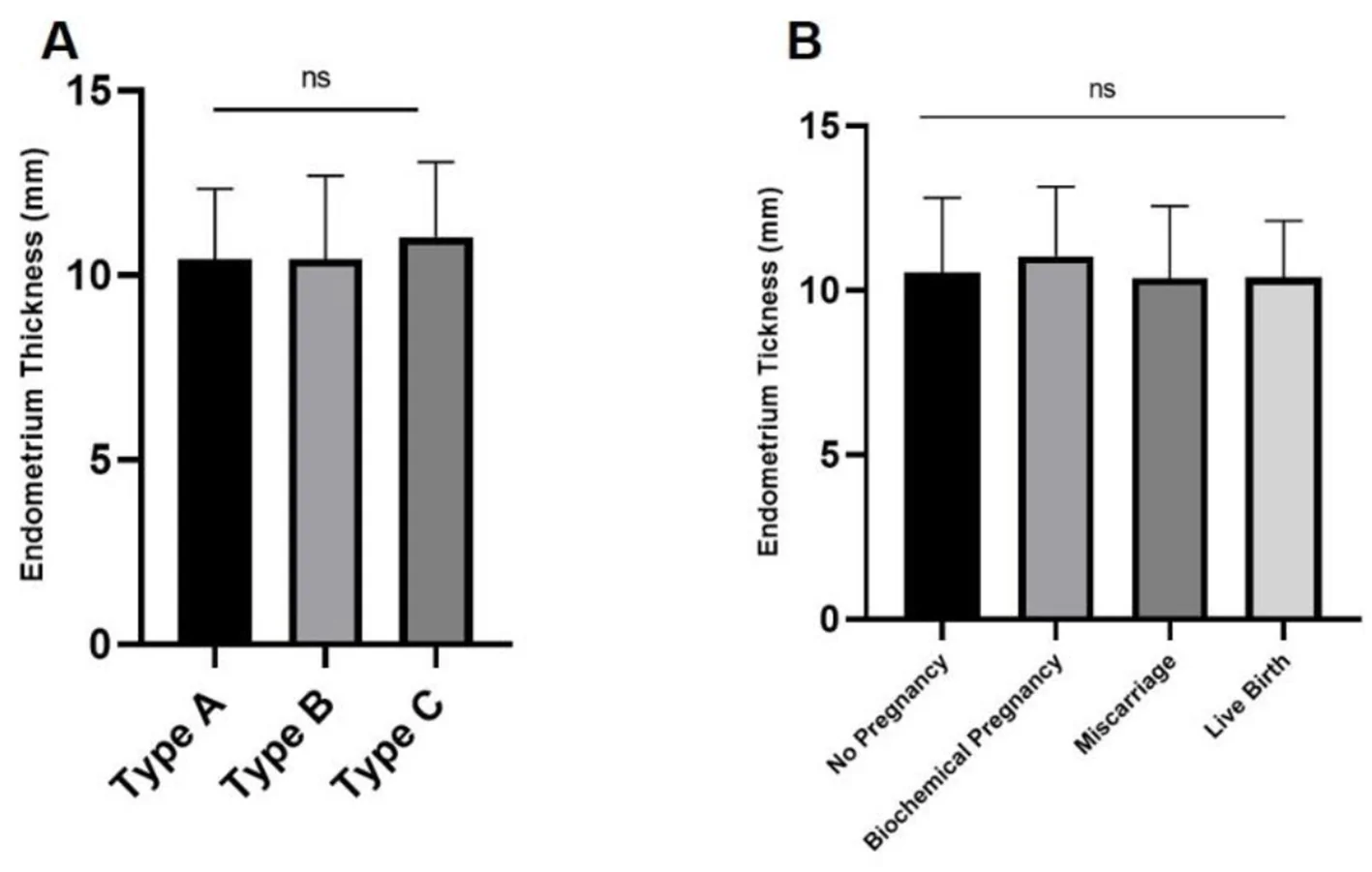
Endometrial thickness according to the endometrial pattern types and pregnancy outcome groups. **(A)** The distribution of endometrial thickness measurements among Type A, Type B, and Type C endometria. **(B)** Endometrial thickness across four pregnancy outcome categories: pregnancy absence, biochemical pregnancy, miscarriage, and live birth. ns: not significant.

The comparative distribution of endometrial thickness in terms of both endometrial pattern types and pregnancy outcomes were given in Figure 3.

Figure 3A shows that endometrial thickness measured in Type A-B and C endometria were similar levels and there was no statistically significant difference between the three groups (*p* = 0.3819). Figure 3B shows that endometrial thickness has similar median values in pregnancy absence, biochemical pregnancy, miscarriage, and live birth outcomes, and that there is no significant difference between these groups (*p* = 0.5676).

**Figure 4:**
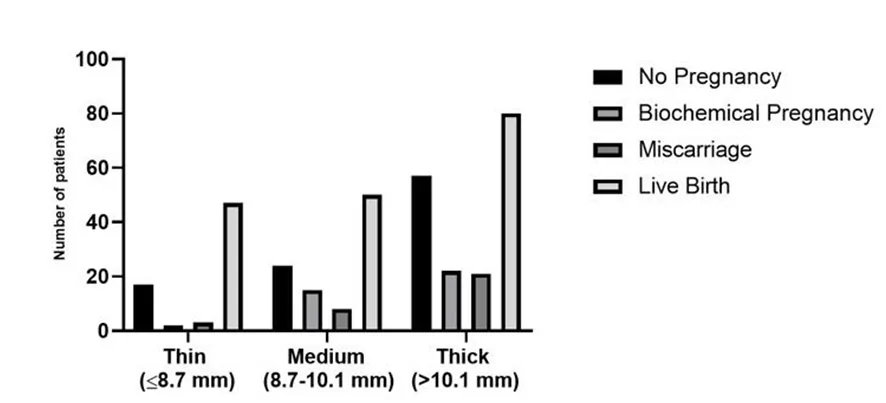
Distribution of pregnancy outcomes across endometrial thickness categories. Endometrial thickness was categorized as thin (<8.7 mm), medium (8.7–10.1 mm), and thick (>10.1 mm).

The relationship between endometrial thickness categories and the distribution of pregnancy outcomes was examined (Figure 4). Endometrial thickness was divided into three categories based on the sample distribution: ‘thin' (< 8.7 mm), ‘medium' (8.7–10.1 mm), and ‘thick' (>10.1 mm). Categories were defined based on the 33^rd^ and 66^th^ percentile values (P_33_ = 8.7 mm, P_66_ = 10.1 mm) (Figure 4).

A significant difference in the distribution of pregnancy failure, biochemical pregnancy, miscarriage, and live birth outcomes was observed between categories (χ^2^ (6) = 15.93, *p* = 0.014). The graph shows that live birth rates are significantly higher, especially in cases with endometrial thickness >10.1 mm, whereas pregnancy failure and abortion rates increase in patients with < 8.7 mm. Additionally, of the 123 patients in the thick endometrial group, 91 (74.0%) had Type A endometrial pattern. These findings support that increased endometrial thickness is positively associated with pregnancy success and the likelihood of live birth.

**Figure 5:**
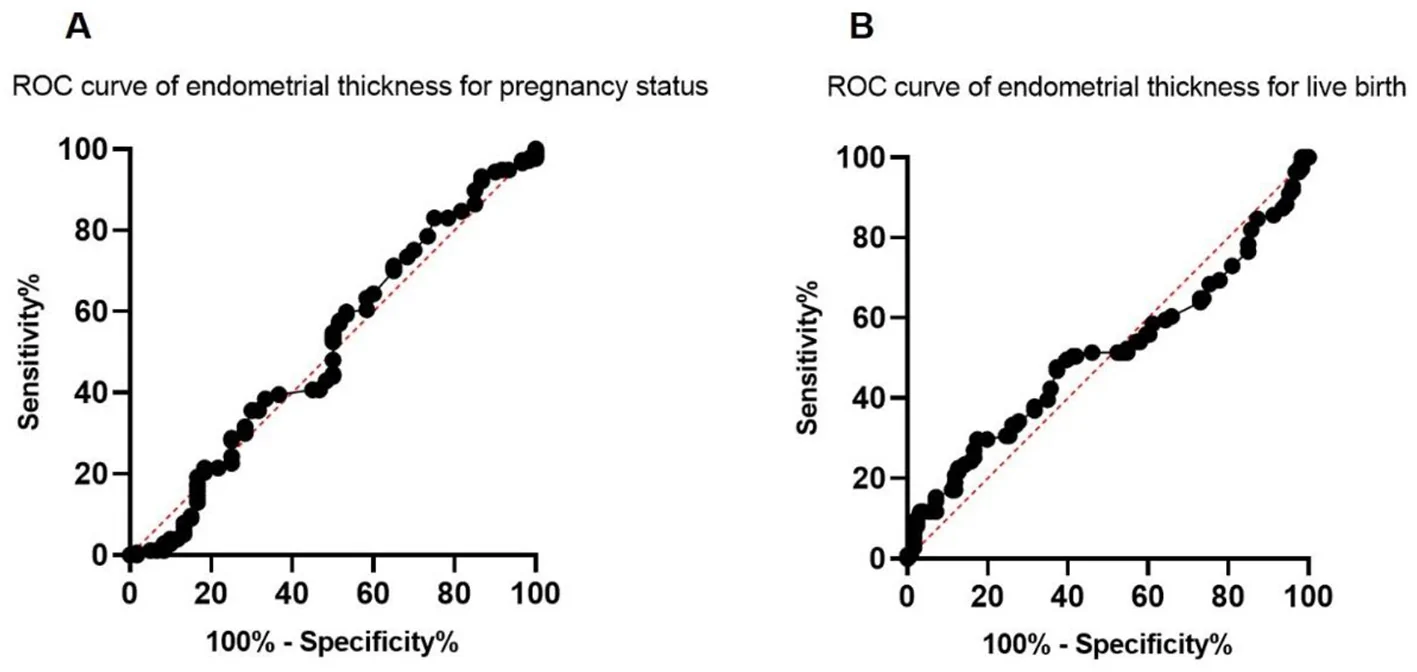
ROC curve analyses evaluating the predictive value of endometrial thickness for **(A)** pregnancy status and **(B)** live birth. AUC values, cut-off points, sensitivity, and specificity are indicated.

ROC analyses were performed to evaluate the predictive power of endometrial thickness on achieving pregnancy and reaching live birth (Figure 5).

In both analyses, the area under the curve (AUC) remained around 0.50, indicating that endometrial thickness did not perform better than random guessing in distinguishing pregnancy status and live birth. In Figure 5A, the AUC for pregnancy formation is 0.505, and although the optimal cut-off value is 8.75 mm, it has no clinical significance due to low sensitivity (25%) and limited specificity (83%). Similarly, in Figure 5B, the AUC for live birth was 0.5103, and the cut-off value of >11.85 mm was insufficient in terms of sensitivity and specificity. These findings indicate that endometrial thickness is not a clinically meaningful biomarker for predicting pregnancy success in the study population.

To evaluate hormonal differences between endometrial patterns, serum E_2_ and P_4_ levels measured during the menstrual cycle and on the day of endometrial assessment were compared between Type A, Type B, and Type C groups. This analysis was performed to determine whether endometrial morphology is associated with hormonal profile and whether specific patterns carry distinct endocrine characteristics (Table 3).

**Table 3:** Serum estradiol (E_2_) and progesterone (P_4_) levels measured during menstruation and on the day of endometrial assessment according to endometrial pattern types (Type A, Type B, and Type C).

Hormone (unit)	Period	Type A (*n* = 177)	Type B (*n* = 37)	Type C (*n* = 23)	*p*–value^*^
**E**_**2**_ **(pg/ml)**	Menstruation	14.0 (2.0–109.0)	10.7 (4.0–64.0)	10.0 (5.0–47.0)	0.3819
	The day of endometrial assessment	273.0 (150.0–1,715.0)	261.0 (151.0–1,428.0)	240.0 (175.0–465)	0.2646
**P**_**4**_ **(ng/ml)**	Menstruation	0.2 (0.02–2)	0.16 (0.01–0.9)	0.10 (0.05–0.88)	0.0799
	The day of endometrial assessment	0.1 (0.01–0.9)	0.136 (0.05–0.5)	0.09 (0.05–0.6)	0.2928

^*^Values are presented as median (min–max). Comparisons between groups were performed using the Kruskal–Wallis test.

There was no significant difference between the groups for either hormone during the menstrual period or on the day of endometrial measurement. The median E_2_ values during the menstrual period were 14.0, 10.7, and 10.0 pg/ml for Type A, B, and C, respectively, and the difference was not statistically significant (*p* = 0.3819). Similarly, endometrial measurement day E_2_ levels showed a similar distribution among the three groups (*p* = 0.2646). P_4_ values measured during the menstrual period appeared higher in Type A, but this difference did not reach significance (*p* = 0.0799). P_4_ levels on the day of endometrial measurement also did not differ between groups (*p* = 0.2928). These findings indicate that serum E_2_ and P_4_ levels are not associated with endometrial pattern classification and that the hormonal profile is not a decisive variable in type differentiation.

**Figure 6:**
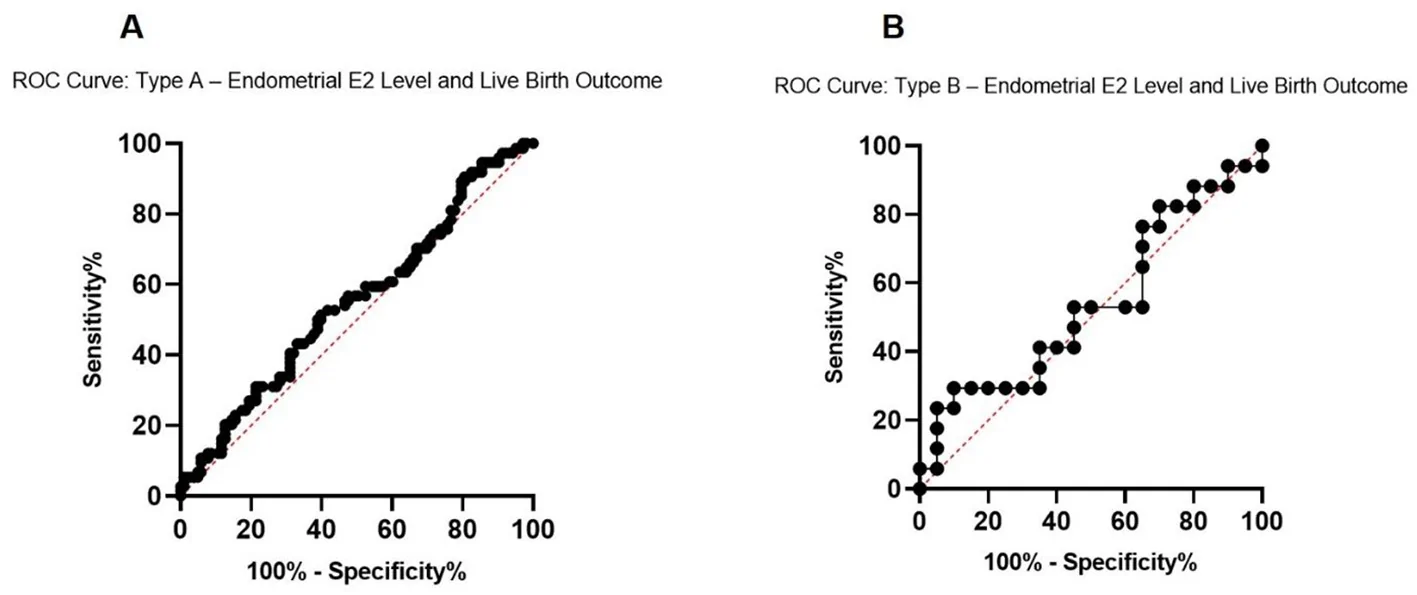
ROC curve analyses assessing the predictive value of serum estradiol (E_2_) levels on endometrial assessment day for live birth in **(A)** Type A and **(B)** Type B endometrial patterns. AUC values and optimal thresholds are shown for each pattern type.

Figure 6 shows the ROC analyses evaluating the performance of serum E_2_ levels measured on endometrial day in patients with Type A and Type B endometrial patterns in predicting live birth outcomes. The analysis was not performed for Type C due to insufficient results.

AUC values were still approximately 0.50 in both pattern groups indicate that serum E_2_ levels do not have significant discriminatory power in distinguishing live births (Type A: AUC = 0.5505 (Figure 6A); Type B: AUC = 0.5382 (Figure 6B). Although the optimal cut-off values were 279.5 pg/ml for Type A and 369.5 pg/ml for Type B, these thresholds showed both low sensitivity and limited clinical applicability.

## Discussion

Endometrial receptivity is one of the most critical components of a successful implantation process, and the usefulness of ultrasonographic endometrial thickness and pattern in predicting pregnancy outcomes has long been debated (1, 6). The literature presents inconsistent findings regarding whether endometrial morphology and pre-transfer hormonal parameters affect IVF-ET success (7, 8). Therefore, in this study, we aimed to re-evaluate the relationship between endometrial pattern types, endometrial thickness, and serum E_2_ and P_4_ levels measured on the day of evaluation with pregnancy, miscarriage, and live birth outcomes in HRT-FET cycles. Our findings suggest that endometrial pattern types may be associated with different clinical outcomes, whereas endometrial thickness and serum E_2_ levels have limited predictive value. Thus, our results show the heterogeneity in the current literature and indicate that the role of these parameters in clinical decision-making processes needs to be reconsidered.

Based on the results obtained in our study, when evaluating the relationship between endometrial morphology and reproductive outcomes, we found that endometrial pattern types were significantly associated with pregnancy, miscarriage, and live birth rates. The group with Type A pattern had the highest live birth rates, while Type C was mostly associated with negative outcomes, which is consistent with FET-based studies suggesting that a trilaminar appearance may be advantageous for implantation. In a comprehensive study conducted by Kuramoto et al. (4) the trilaminar (Type A) pattern was found to be associated with the highest live birth rate, while a low pregnancy rate was established in patients with the homogeneous hyperechoic (Type C) pattern, with live birth rates decreasing from about 56% in the most distinct trilaminar group to around 15% in non-trilaminar cases and miscarriage approaching 47% in the latter, as supporting our findings. Although many studies have obtained results parallel to this current study (3, 5), there are also studies with conflicting findings. For instance, in a large retrospective cohort study conducted by Cheng et al. on FET cycles, live birth rates were found to be significantly lower in endometrium that still maintained a trilaminar appearance on the day of embryo transfer. Homogenized non-trilaminar patterns, i.e., Type C, were associated with higher live birth rates compared to Type A, where Type A showed a live birth rate of only about 19%, while non-trilinear patterns had significantly higher live birth rates and lower miscarriage risk (9). A relatively older study reported that the endometrial pattern was not a predictive factor for live birth and/or clinical pregnancy rates, and that the trilaminar appearance did not provide a significant advantage over non-trilaminar patterns (10). Although our findings and those of Kuramoto et al. (4) consistently demonstrate poorer outcomes in non-trilaminar endometrial patterns, the biological mechanisms underlying this association remain unknown. Kuramoto et al. suggested that endometrial hyperechogenicity may be linked to progesterone-related changes and that fibrotic alterations following intrauterine procedures could contribute to non-trilaminar profiles. Notably, some patients with non-trilaminar patterns in their study improved to a partial trilaminar appearance following hysteroscopy and treatment for chronic endometritis, resulting in subsequent live births (4). This observation raises the possibility that subclinical endometrial inflammation may also play a role in the development of unfavorable endometrial patterns. However, immunohistopathological and microbiological confirmation of these hypotheses is currently lacking, and further studies are warranted. To sum up, it can be interpreted that morphological endometrial pattern types are insufficient to explain receptivity in clinical translation, and pattern-based classifications may not carry similar prognostic value across all patient groups.

Our findings that endometrial thickness cannot distinguish between pregnancy, miscarriage, or live birth outcomes is consistent with large-sample studies reporting that thickness above a certain cut-off value does not carry predictive value (11, 12). When Mahutte and colleagues compared fresh and frozen cycles, they noted that live birth rates remained stable after endometrial thickness reached 7–10 mm in FET cycles; this improvement was independent of the patient's age, timing of embryo transfer, or the number of eggs retrieved during egg retrieval (11). Additionally, while some large FET cohorts have demonstrated better outcomes with increasing endometrial thickness, their results show that this effect is most noticeable in the very thin endometrium range (< 7 mm) and tends to plateau thereafter; this pattern is consistent with the ROC analyses of our study showing no discriminative value (13). Although the categorical analysis of endometrial thickness demonstrated a significant association between the thick category (>10.1 mm) and higher live birth rates (*p* = 0.014), ROC analysis revealed no discriminatory power for endometrial thickness as a continuous variable (AUC ≈ 0.50). This apparent discrepancy may be explained by the uneven distribution of endometrial pattern types across thickness categories; 74% of the patients in the “thick” group had Type A endometrial pattern, which was independently associated with the highest live birth rates. Therefore, the favorable outcomes observed in the thick category likely reflect the predominance of Type A pattern rather than a true threshold effect of endometrial thickness.

According to our results, the serum estradiol levels measured on the day of endometrial assessment did not demonstrate the ability to predict live birth, and the AUC values remained at a random level (lacking discriminatory power). This supports studies indicating that E_2_ levels in HRT-FET cycles have limited clinical value (14). The findings of Li et al., showing that serum E_2_ levels are not associated with pregnancy outcomes in HRT-FET cycles with blastocyst transfer, support the random ROC performance obtained for E_2_ in our study; in the study, only E_2_ levels above 413.6 pg/ml in cleavage-stage transfers were associated with negative outcomes (15). From another perspective, while some studies suggest that excessively high estradiol levels may negatively affect receptivity (16), some meta-analyses support this idea but show that this relationship is quite weak and clinically insignificant (17).

Two studies led by Zhou and Goldman consistently demonstrated significant decreases in live birth and implantation rates in patients with high E_2_ levels. In detail, focusing on the study of Zhou et al., they found that live birth rates decreased when E_2_ levels were 400 pg/ml or higher. Consistent with these findings, a systematic review and meta-analysis covering more than 16.000 patients recommends keeping E_2_ levels < 500 pg/ml for successful outcomes. The meta-analysis indicated that levels up to 200 pg/ml yielded the highest success for clinical pregnancy, while the optimal range for live birth was 200 to 500 pg/ml. The common conclusion is that serum E_2_ concentrations prior to P_4_ initiation must be carefully controlled to achieve the best FET outcomes (14, 16, 17). Additionally, the lowest miscarriage rate (6%) was observed in patients with an E_2_ level of 1,000–2,000 pg/ml (17). Although the data obtained provide strong evidence from large retrospective studies that high E_2_ levels (especially those above the 400–500 pg/ml range) reduce the live birth rate, there is general uncertainty due to the existence of studies in the literature that do not observe this relationship or report different optimal threshold values (14, 16, 18, 19).

This study has several limitations that should be acknowledged. Firstly, the retrospective design inherently introduces the risk of selection bias and limits causal inference. Secondly, the uneven distribution of patients across endometrial pattern groups, particularly the small sample size in the Type C group may compromise statistical power and limits the generalizability of subgroup findings. This imbalance also precluded ROC analysis for Type C. Thirdly, although maternal age was adjusted for in the logistic regression model, data on infertility etiology and gravidity/parity were not available, which limits the assessment of potential confounders related to reproductive history. In addition, endometrial pattern assessment is inherently subjective, and inter-observer variability was not evaluated in this study. Fifth, although BMI did not differ significantly between groups, it was not included as a confounder in the regression model. Finally, serum progesterone levels on the day of embryo transfer were not analyzed as a primary variable, although cycles with P_4_ levels < 10 ng/ml were canceled per protocol.

In summary, our study demonstrates that endometrial pattern may be associated with clinical outcomes, whereas endometrial thickness and serum E_2_ levels retain low predictive value and discriminatory power. These findings reflect the heterogeneity in the existing literature and suggest that the role of these parameters in clinical decision-making processes during HRT-FET cycles should be re-evaluated.

## Data Availability

The original contributions presented in the study are included in the article/supplementary material, further inquiries can be directed to the corresponding author.
